# A Computational Approach for the Discovery of Novel DNA Methyltransferase Inhibitors

**DOI:** 10.3390/cimb46040213

**Published:** 2024-04-16

**Authors:** Eftichia Kritsi, Paris Christodoulou, Thalia Tsiaka, Panagiotis Georgiadis, Maria Zervou

**Affiliations:** Institute of Chemical Biology, National Hellenic Research Foundation, 48 Vassileos Constantinou Avenue, 11635 Athens, Greece; pchristodoulou@eie.gr (P.C.); thtsiaka@eie.gr (T.T.); panosg@eie.gr (P.G.)

**Keywords:** epigenetics, DNA methyltransferases, DNMT inhibitors, natural-derived chemo-libraries, virtual screening, pharmacophore modeling

## Abstract

Nowadays, the explosion of knowledge in the field of epigenetics has revealed new pathways toward the treatment of multifactorial diseases, rendering the key players of the epigenetic machinery the focus of today’s pharmaceutical landscape. Among epigenetic enzymes, DNA methyltransferases (DNMTs) are first studied as inhibition targets for cancer treatment. The increasing clinical interest in DNMTs has led to advanced experimental and computational strategies in the search for novel DNMT inhibitors. Considering the importance of epigenetic targets as a novel and promising pharmaceutical trend, the present study attempted to discover novel inhibitors of natural origin against DNMTs using a combination of structure and ligand-based computational approaches. Particularly, a pharmacophore-based virtual screening was performed, followed by molecular docking and molecular dynamics simulations in order to establish an accurate and robust selection methodology. Our screening protocol prioritized five natural-derived compounds, derivatives of coumarins, flavones, chalcones, benzoic acids, and phenazine, bearing completely diverse chemical scaffolds from FDA-approved “Epi-drugs”. Their total DNMT inhibitory activity was evaluated, revealing promising results for the derived hits with an inhibitory activity ranging within 30–45% at 100 µM of the tested compounds.

## 1. Introduction

Nowadays, epigenetics is one of the most promising and expanding fields in the pharmaceutical landscape. The term epigenetics is defined as the heritable, reversible modulation of gene expression that occurs without changes in the underlying DNA sequence [1]. The dynamic regulation of epigenetic modifications causes different functional outcomes that play a crucial role in biological procedures [2]. Epigenetic dysfunction is tightly connected with the pathogenesis and progression of a plethora of diseases, involving multifactorial diseases. Specifically, cancers, chronic diseases, neurodegenerative disorders, and diabetes highlight the crucial role of these covalent modifications [3,4,5].

Very few epigenetic targets have been examined so far in different stages of the drug discovery process. Only seven epigenetic drugs have been approved by FDA, targeting DNMT, HDAC, or EZH2 epigenetic enzymes, all being drugs for malignancies. The application of epigenetic drugs for the treatment of other multifactorial diseases remains unexplored [6].

Epigenetic re-programming through suitable small molecules could alter a plethora of cellular pathways, allowing, for instance, the manipulation of pathways previously thought to be undruggable [7,8]. It is therefore apparent that small molecule inhibitors of the key epigenetic enzymes (epigenetic targets) will not only provide highly useful chemical tools to further characterize these vitally important mechanisms but also provide chemical starting points for the development of novel epigenetic therapeutic approaches [9]. Consequently, the discovery of small molecule inhibitors against these epigenetic targets has become the focus of today’s research.

The epigenetic target inhibitors were discovered mainly through high-throughput screening, random screening, and biophysical screening approaches. However, these wet experimental methods usually suffer from a high cost and a comparatively low hit rate [10]. Computational techniques and methodologies as a rapid and economic strategy have been widely used in the medicinal chemistry area for hit/lead discovery [11]. Several newly emerging in silico approaches could be utilized to identify epigenetic target inhibitors more efficiently [12].

DNA methyltransferases are among the first studied enzymes as inhibition targets for cancer treatment. Over the past few years, more therapeutic opportunities related to the modulation of DNMT activity have been emerging [13], rendering them extremely promising targets. The increasing clinical interest in DNMTs has led to advanced experimental and computational strategies in the search for novel DNMT inhibitors [14].

Virtual screening has emerged as a substantial research tool in drug discovery and is defined as a set of computational methods that analyze large databases of compounds to identify potential hit candidates [15]. A remarkable number of pharmaceutical companies and academia utilize these methods, worldwide, highlighting their contribution to the drug design process [16].

In the field of the discovery of epigenetic target inhibitors, in silico methods are utilized as an indispensable tool and constitute the current research hotspot [17]. The crystal structures of human DNMT enzymes have created new perspectives to build up potent in silico screening approaches to reveal novel specific epigenetic inhibitors [18].

Natural compounds are widely known for their pharmaceutical properties and they have been presumed to be safer compared to synthetic. They possess enormous structural and chemical diversity and continue to inspire novel discoveries in pharmaceutical chemistry [19]. They can also regulate gene expression via epigenetic mechanisms and have gained considerable importance owing to their demonstrated ability to suppress cancers [20]. According to the literature, several polyphenolic compounds are proven to possess epigenetic inhibition activity, including resveratrol, curcumin, and epigallocatechin gallate [21,22,23]. Although the biodiversity of natural compounds has been poorly explored, research suggests that they can offer unlimited expectations in the field of epigenetic drug design [24].

Towards this direction, the present study aimed at the discovery of novel inhibitors of natural origin against DNMT isoforms using a combined methodology, including structure- and ligand-based computational approaches. For the present scope, a series of commercially available natural compounds chemo-libraries were virtually screened against our generated pharmacophore hypotheses and the retrieved compounds were further subjected to molecular modeling studies into the catalytic binding site of DNMT1. A series of natural compounds that differentiate from “Epi-drugs” were prioritized for supply and further evaluation for their DNMT inhibitory activity. The results clearly indicated that all examined compounds exhibit DNMT inhibitory activity and could serve as new starting scaffolds for further hit-to-lead optimization.

## 2. Materials and Methods

### 2.1. Pharmacophore Model Generation and Validation

For the generation and validation of the ligand-based pharmacophore model, LigandScout 4.0 Advanced software was used (InteLigand, GmbH, Vienna, Austria) [25].

The creation of the pharmacophore model was based on the chemical scaffolds of FDA DNMT-approved drugs and inhibitors in clinical studies. For this scope, the Human Epigenetic Drug Database (HEDD) [26] (http://hedds.org/, accessed on 16 December 2021) and the Human Epigenetic Enzyme and Modulator Database (HEMD) [27] (https://mdl.shsmu.edu.cn/HEMD/, accessed on 16 December 2021) were utilized as a pool of compounds. Particularly, the training set contained seven commercially available epigenetic inhibitors (Procainamide, Bobcat339, CM272, SGI-1027, Thioguanine, RG108, and Decitabine), while the test set included six known epigenetic inhibitors with low uM DNMT inhibitory activity. The chemical structures and epigenetic activity of training and test set compounds are presented in Tables S1 and S2 (Supplementary Materials), respectively.

In continuation, all compounds were sketched and were minimized in the MAESTRO interface [28]. Then, they were prepared at pH 7.0 ± 0.5 using the LigPrep module [29] of the MAESTRO interface [28].

The ligand set conformers were generated using OMEGA [30] and the maximum number of conformations per ligand was set as equal to 50. The conformers were clustered according to the pharmacophore alignment score leading to the creation of 10 pharmacophore hypotheses, ranked according to the Pharmacophore-Fit score. The applied scoring function was the “Pharmacophore Alignment Score” and the selected pharmacophore type was the ‘‘merged” pharmacophore features.

For the pharmacophore model validation, three compound libraries were constructed. The first library included ninety-one active compounds with inhibitory activity against the DNMT (DNMT1 and DNMT3A) family of enzymes (10 nM < IC_50_ < 500 µM), and the second fourteen inactive compounds (IC_50_ > 1 mM); due to the small number of inactive compounds available in the literature, a decoy library was also created, consisting of 4849 compounds structurally similar to the active ones, but experimentally not tested for biological activity. For the generation of the decoy’s library, DUD-E [31] (https://dude.docking.org/generate, accessed on 26 January 2022), a freely available tool, was used.

The pharmacophore model assessment was conducted using classic descriptors, such as Sensitivity (Se), Specificity (Sp), Enrichment Factor (EF), and the Receiver Operating Characteristic (ROC) curve.

### 2.2. Virtual Screening

#### 2.2.1. Pharmacophore-Based Virtual Screening

Pharmacophore-based virtual screening was implemented in a series of commercially available natural compounds libraries (Ambinter—http://www.ambinter.com/, Specs—https://www.specs.net/, Indofine—https://indofinechemical.com/, InterBioScreen—https:www.ibscreen.com/, Selleckchem—https://www.selleckchem.com/, Analyticon Discovery—https://ac-discovery.com/, PhyProof—https://www.phytolab.com/en/our-services/reference-substances-phyproof/, MolPort—https://www.molport.com/, Enamine—https://enamine.net/, Nubbe—https://nubbe.iq.unesp.br/portal/nubbe-search.html — accessed on 5 February 2022), containing more than 250,000 natural compounds. The retrieved pool of compounds was converted to an appropriate database using the idbgen tool of LigandScout [32]. The database generation was performed using OMEGA [30] fast settings and 25 conformers per compound were calculated.

The hits were filtered based on the higher pharmacophore-fit score values (Pharmacophore Fit Score ranged from 42 to 46). The derived hits were further filtered via Knime, an open-source platform (https://www.knime.com/knime-analytics-platform—accessed on 10 April 2022) according to the physicochemical properties of the FDA-approved epigenetic drugs, estimated by Qikprop [33] and Canvas [34] modules of MAESTRO [28]. Specifically, the filtering criteria were the following: lipophilicity (AlogP) value, molecular weight (MW), number of hydrogen bond acceptors (HBA), number of hydrogen donors (HBD), number of rotatable bonds (RB), and polar surface area (PSA) value for the FDA-approved epigenetic drugs (Table S3, Supplementary Materials) and the modified drug-likeness values were set as follows: −2 ≤ AlogP ≤ 5, 150 ≤ MW ≤ 650, 1 ≤ HBA ≤ 12, 2 ≤ HBD ≤ 10, 1 ≤ RB ≤ 20 and 50 ≤ PSA ≤ 250 Å^2^. All compounds were studied under physiological conditions.

#### 2.2.2. Molecular Docking Studies

The compounds that passed the pharmacophore and physicochemical filtering were subjected to further processing through molecular docking studies into the Sinefungin binding site of human DNMT1 (PDB: 3SWR, 2.49 Å) isoform. The selection of 3SWR crystal structure was based on the fact that the *h*DNMT1 enzyme is co-crystallized with Sinefungin, which is reported as a potent DNMT inhibitor [35] and it could be of interest as a model for inhibitory interactions [18,36]. The retrieved crystal structure was prepared by the Protein Preparation Wizard [37]. In particular, all missing residues and hydrogen atoms were added and bond orders were assigned and then minimized using the OPLS3 force field.

All hits derived from our screening pipeline were prepared at pH = 7.0 ± 0.5 by applying LigPrep [29] while the original state was also included and was docked against the *h*DNMT1 catalytic site by implementing the Glide module [38] in standard precision (SP) and in extra precision (XP) mode as well as the Induced-Fit protocol [39] through the Maestro interface [28]. The grid box was created based on the centroid of the workspace ligand (Sinefungin) with Site advanced settings to keep the ligand diameter midpoint box within dimensions 10 × 10 × 10 Å^3^, and the maximum number of poses was set as equal to 10. All generated poses were visually inspected and analyzed. Also, for validation reasons, the Pose Viewer and Pose Filter tools of the Schrodinger interface were applied. The total energy of the *h*DNMT1-compounds complexes and the contributions to the total energy are reported using prime MM-GBSA.

#### 2.2.3. Molecular Dynamics Simulations

All Molecular Dynamics (MD) simulations of the prioritized hits against the *h*DNMT1 were performed using Desmond software (https://www.schrodinger.com/platform/products/maestro/) [40]. The bound complexes were inserted in an orthorhombic box containing ~35,000 water molecules, the TIP4P was used as a solvent model, the box size calculation method was buffer, and the buffer distances were all set to 10. A OPLS3 force field was applied and the systems were neutralized by adding 15 Na^+^ and Cl^−1^ ions. Modeled systems were relaxed (relaxation time = 10 ns) and subsequently were subjected to 50 ns MD simulations. A time step of 2 fs was used for the integration of equations of motion. The ensemble class was NPT, maintaining the temperature and the pressure equal to 300 K and 1.013 bar, respectively. The thermostat method was Nose–Hoover chain and the barostat method was Martyna–Tobias–Klein. Also, the relaxation time was defined as equal to 1.0 ps and 2.0 ps, respectively.

#### 2.2.4. ADMET Properties

The ADMET properties of the potential inhibitors were predicted by applying ADMETlab 2.0 (https://admetmesh.scbdd.com/—accessed on 1 March 2024) [41].

### 2.3. Total DNMT Inhibitory Activity Evaluation

#### DNMT Inhibition Measurements

DNMT inhibition was evaluated by Abcam DNMT activity assay kit (https://www.abcam.com/en-gr/products/assay-kits/dnmt-activity-assay-kit-colorimetric-ab113467, last accessed 30 April 2022) according to manufacturer’s instructions. Briefly, 1 uL of 50 mg/mL DNMT solution was added in every well. Then, 5 µL of the tested compounds in a final concentration of 100 µM were incubated in duplicates at 37 °C for 90 min. Wells containing the studied enzyme were used as controls for the baseline DNMT activity in the absence of an inhibitor. The inhibition of 50 ng DNMT was analyzed by 450 nm ELISA in a TECAN microplate spectrophotometer. Finally, the DNMT inhibition of each sample was calculated by the kit manufacturer’s suggested formula. A Tukey *t*-test was performed to evaluate the statistical significance of the results.

## 3. Results

**Figure 1:**
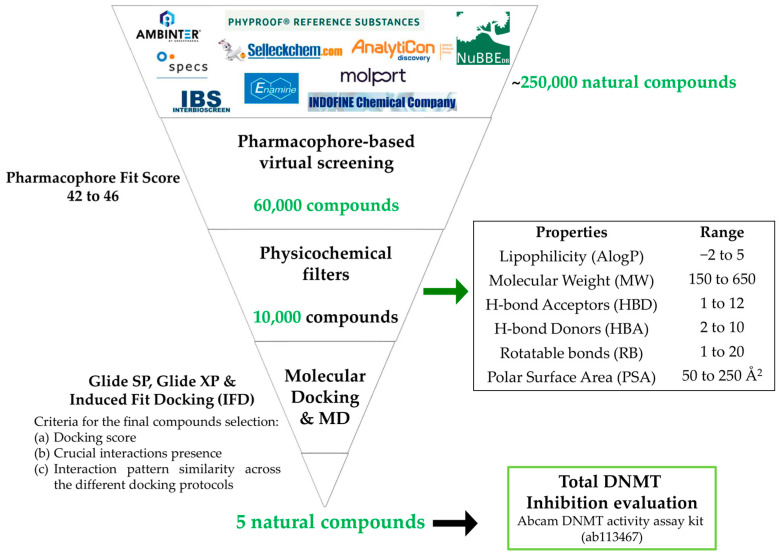
Virtual screening protocol workflow.

The virtual screening flowchart of the present study is illustrated in Figure 1.

### 3.1. Pharmacophore Model

#### 3.1.1. Ligand-Based Pharmacophore Model Generation

A dataset of thirteen chemically diverse epigenetic inhibitors retrieved from the HEDD [26] (http://hedds.org/, accessed on 16 December 2021) and the HEMD [27] (https://mdl.shsmu.edu.cn/HEMD/, accessed on 16 December 2021) databases was selected for the generation of the ligand-based pharmacophore model. Specifically, seven commercially available epigenetic inhibitors, including three nucleoside and four non-nucleoside analogs, constituted the training set (Table S1, Supplementary Materials), and six known epigenetic inhibitors with low uM DNMT1 inhibitory activity, bearing a variety of structural features, composed the test set (Table S2, Supplementary Materials).

**Figure 2:**
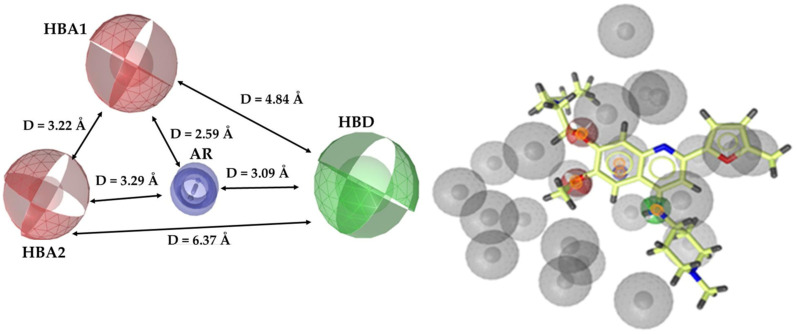
(**Left**) The features of the optimum pharmacophore model; (**right**) CM272 DNMT inhibitor (yellow) fitted on the optimum pharmacophore model. The features are depicted with the following color coding: hydrogen bond acceptors (HBA) as red spheres, the hydrogen bond donor (HBD) as a green sphere, the aromatic ring (AR) as a blue ring, and exclusion volumes (Ex. Vol.) as gray spheres. The distances (Å) between the chemical features are illustrated as black lines. The figure was created with LigandScout 4.0 Advanced from InteLigand [25].

Initially, a series of ten pharmacophore hypotheses were produced and their fit to pharmacophore features was evaluated. The results analysis indicated that for the top-ranking hypothesis, all the examined compounds consisted of four common pharmacophore features. Particularly, the pharmacophore model possessed two hydrogen bond acceptors (HBA), one hydrogen bond donor (HBD), one aromatic ring (AR), and twenty-nine exclusion volumes. Subsequently, the derived model was optimized by increasing the volume of HBA and HBD, decreasing the volume of AR, and also by reducing the number of exclusion volumes to nineteen and modifying their size according to the alignment of the molecules of the training set. The optimized pharmacophore features and the fit of the CM272 DNMT inhibitor to the optimum model are presented in Figure 2.

#### 3.1.2. Ligand-Based Pharmacophore Model Validation

Subsequently, the resulting model was subjected to a validation process in order to assess its ability to select as many biologically active compounds from a structurally diverse compound database as possible and discard most of the inactive compounds [42]. For the abovementioned scope, three different sets of compounds were established, a set of actives, a set of inactives, and a decoy set since the number of inactive compounds available in the literature was inadequate for the study.

**Figure 3:**
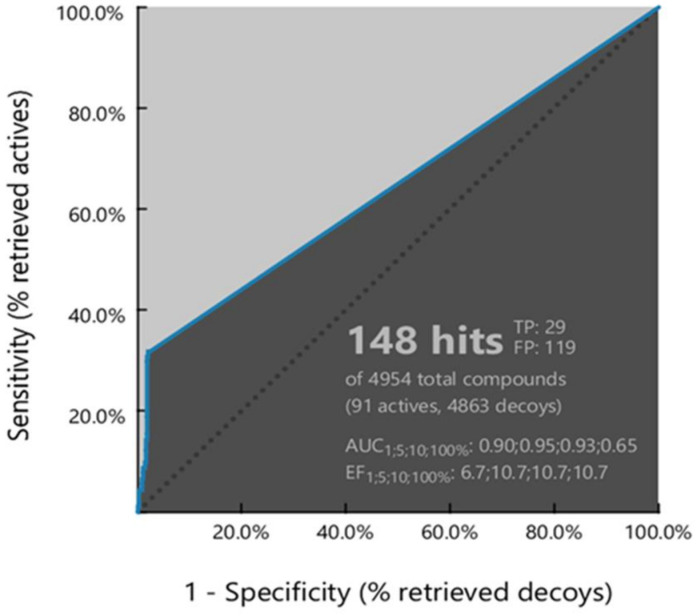
Illustration of the receiver operating characteristic (ROC) curve of the optimized pharmacophore model. The figure was generated with LigandScout 4.0 Advanced from InteLigand [25].

The ability of the derived model to correctly classify the list of compounds as actives or inactive was examined via Receiver Operating Characteristic (ROC) curve analysis (Figure 3). Additionally, Area Under Curve (AUC), Sensitivity (Se), Specificity (Sp), and Enrichment Factor (EF), quantitative key parameters [42,43], were exploited to confirm the validity of the model (Table 1). Finally, the validation of the model was completed by calculating statistical significance variables (Table 1).

The evaluation of the ROC curve, as depicted in Figure 3, clearly demonstrates that the computed model exhibits a good selection score and is certainly better than random selection (AUC = 0.65 > 0.5). Notably, the curve presents a steep slope during the initial stages of screening, indicating a high enrichment of actives among the top-ranked hit list compounds. This observation is further corroborated by the values of sensitivity (Se = 0.32) and false positive rate (1-Sp = 0.02) along with the enrichment factor (EF = 10.7), elucidated in Table 1. Additionally, the reliability of the model was evidenced by its ability to successfully recover almost 35% of the active compounds while the percentage of retrieved inactives and decoys remains significantly lower compared to actives (Table 1).

In consideration of the abovementioned outcomes, the generated model was regarded as a reliable virtual screening filter.

### 3.2. Virtual Screening (VS) Results

#### 3.2.1. Pharmacophore-Based Virtual Screening

The in-site generated database of ~250,000 natural compounds, derived from a series of different chemo-libraries, was screened according to the obtained pharmacophore model features. The screening results revealed that in total 60,000 natural compounds were fitted to the generated pharmacophore model features. Subsequently, the hits with the top-ranked pharmacophore fit score (Pharmacophore-Fit score range from 44 to 46) were subjected to further filtering based on modified drug-likeness values of the FDA-approved epigenetic drugs (Table S3, Supplementary Materials), resulting in a selection of 10,000 natural compounds.

#### 3.2.2. Molecular Docking

In a further step, molecular docking studies were performed on compounds that passed the filtering criteria and possessed the highest fit to the pharmacophore model features. Especially, the selected compounds were docked at the Sinefungin binding site of the *h*DNMT1 (PDB: 3SWR) isoform. In an effort to reinforce the accuracy of the results, a consensus docking protocol was applied, including three different algorithms (Glide SP, Glide-XP, IFD), to predict the binding modes of the examined compounds. The prioritization of the final compounds was mainly based on three criteria: (a) the calculated binding affinity, reflected as docking score, in comparison to the crystal complex ligand, (b) the retention of crucial interactions participating in the Sinefungin binding into the *h*DNMT1 catalytic site, and (c) the interaction pattern similarity across the three different docking protocols (Table S4, Supplementary Materials).

**Figure 4:**
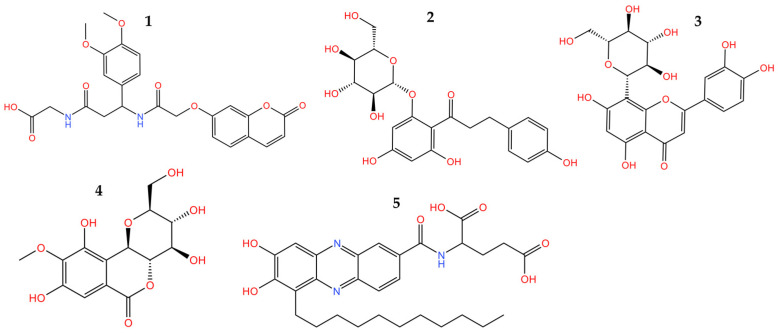
Selected compounds from pharmacophore-based vs. and molecular docking. **1**: 2-(3-(3,4-dimethoxyphenyl)-3-(2-((2-oxo-2H-chromen-7-yl)oxy)acetamido)propanamido)acetic acid, **2**: Phlorizin, **3**: Orientin, **4**: Bergenin, and **5**: 2-[(7,8-dihydroxy-6-undecylphenazin-2-yl)formamido]pentanedioic acid.

**Figure 5:**
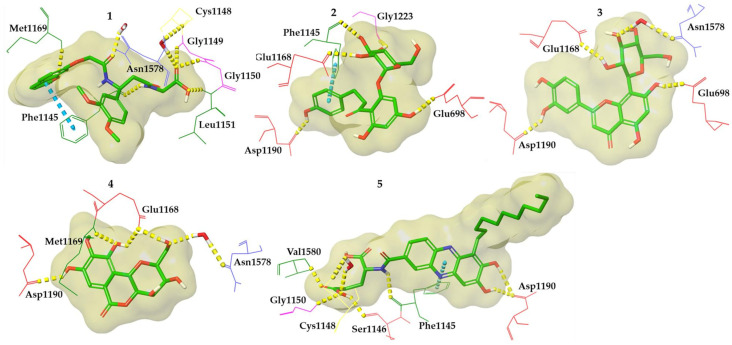
Representative binding poses of the compounds **1**–**5**, derived from Glide-XP. **1**: 2-(3-(3,4-dimethoxyphenyl)-3-(2-((2-oxo-2H-chromen-7-yl)oxy)acetamido)propanamido)acetic acid, **2**: Phlorizin, **3**: Orientin, **4**: Bergenin, and **5**: 2-[(7,8-dihydroxy-6-undecylphenazin-2-yl)formamido]pentanedioic acid. Hydrogen bonds are depicted with yellow dashed lines and pi-pi interactions are illustrated with blue dashed lines.

Taking into consideration the aforementioned criteria, five compounds, bearing completely different chemical scaffolds, were picked and explored further (Figure 4). Representative compounds’ docked poses are depicted in Figure 5. Considering compounds **1** and **5**, their ionized forms present on physiological pH retain the same binding mode as the protonated ones keeping the majority of the crucial interactions (Table S4, Supplementary Materials). The ADMET properties of the five most promising compounds were predicted and illustrated in Table S5 (Supplementary Materials). Also, the binding energy and individual energy terms of *h*DNMT1-selected compound complexes were calculated and presented in Table S6 (Supplementary Materials).

#### 3.2.3. Molecular Dynamics Simulations

In an effort to further evaluate the stability of the interactions developed from the selected compounds into the *h*DNMT1 catalytic site, each molecule was subjected to unconstrained molecular dynamics simulations (t = 50 ns). The conformational stability of the *h*DNMT1-selected compound complexes was evaluated using the root mean square deviation (RMSD) values of potential ligand and enzyme Ca atoms during the entire run (Figure S1, Supplementary Materials). Ligand root mean square fluctuation (RMSF) values were also calculated in order to explore further ligand’s conformational flexibility at the atomic level (Figure S2, Supplementary Materials). From RMSD values, it was observed that the enzyme Ca atoms in the complexes with compounds **1**, **2**, **3**, and **4** converged below 2.0 Å after 10 ns, indicating that the MD trajectories achieved equilibrium [44]. The protein backbone RMSD fluctuation was more prominent in the case of the *h*DNMT1-compound **5** predicted complex succeeding though to converge (<2.0 Å) after 27 ns. Moreover, RMSD values of compounds **1**, **3**, and **4**, remained below 2.0 Å after 10 ns showing stable ligand poses until the end of the MD run. Within the same time frame, higher mobility within the catalytic site was observed for compound **2**, which also underwent a jump after 30 ns, indicating the conformational flexibility of the glycosidic moiety of the molecule as also evidenced by the RMSF values of the sugar atoms. Compound **5** displayed also a higher mobility within the active site of *h*DNMT1 indicative of its alkyl chain’s increased conformational flexibility as also evidenced by the corresponding RMSF plot.

**Figure 6:**
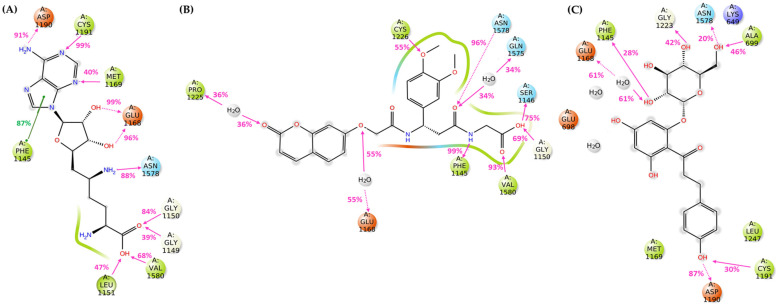

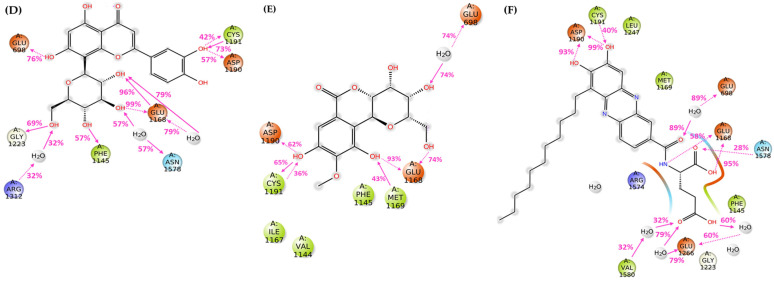
**1**: 2D ligand interaction diagrams of (**A**) Sinefungin and the examined compounds indicating the frequency of occurrence of interactions. (**B**) **1**: 2-(3-(3,4-dimethoxyphenyl)-3-(2-((2-oxo-2H-chromen-7-yl)oxy)acetamido)propanamido)acetic acid, (**C**) **2**: Phlorizin, (**D**) **3**: Orientin, (**E**) **4**: Bergenin, and (**F**) **5**: 2-[(7,8-dihydroxy-6-undecylphenazin-2-yl)formamido]pentanedioic acid. Hydrogen bonds and pi-pi interactions are illustrated with pink and green lines, respectively. The figure was created using Desmond software.

The MD simulation results are depicted in Figure 6, revealing that all compounds are well accommodated in the *h*DNMT1 catalytic site preserving most of the critical interactions within the time course. For comparison, the MD simulation results for Sinefungin bound at the hDNMT1 catalytic site are also presented in Figure 6.

In the case of compound **1**, upon examination of the results in the catalytic center of the *h*DNMT1 isoform, direct hydrogen bonds at high percentages were formed with the critical amino acids Phe1145 (99%), Asn1578 (96%) and Val1580 (93%). Also, a water-mediated hydrogen bond is developed with the crucial amino acid Glu1168 (55%). Additionally, the binding is stabilized through the creation of direct and water-mediated hydrogen bonds with the amino acids Ser1146 (75%), Gly1150 (~70%), Pro1225 (~40%), Cys1226 (55%), and Gln1575 (~35%) (Figure 6).

Molecular dynamics results of compound **2** in the catalytic site of the *h*DNMT1 isoform reveal the formation of direct hydrogen bonds with the critical amino acids Phe1145 (~30%), Asp1190 (~90%), Cys1191 (30%), and Asn1578 (20%), along with a water-mediated hydrogen bond with the also critical amino acid Glu1168 (~60%). Moreover, the binding is stabilized through the development of direct hydrogen bonds Ala699 (~50%) and with Gly1223 (~45%) (Figure 6).

The analysis of the results of compound **3** indicated the formation of interactions with critical amino acids Phe1145 (~60%), Glu1168 (96%/99% and ~80%), Asp1190 (~60%), Cys1191 (~45/~75%), and Asn1578 (~60%). Additionally, hydrogen bonds were formed between the -OH groups of compound **4** and the amino acids Glu698 (~80%), Gly1223 (~70%), and Arg1312 (~35%), further stabilizing the binding (Figure 6).

The critical amino acids Glu1168 (~75%/~95%), Met1169 (~45%), Asp1190 (~65), and Cys1191 (36% and 65%) interact through the formation of direct hydrogen bonds with the pharmacophoric moieties of the compound **4**. Further stabilization of the binding occurs through a water-mediated hydrogen bond with the amino acid Glu698 (~75) (Figure 6).

In the case of compound **5**, it was revealed that hydrogen bonds are created with the critical amino acids Glu1168 (~60% and 95%), Asp1190 (~95% and 99%), Cys1191 (40%), Asn1578 (~30%), and Val1580 (~35%). Furthermore, the binding is further strengthened through the development of water-mediated hydrogen bonds at particularly high percentages with Glu698 (~90%) and Glu1266 (~60% and ~80%) (Figure 6).

#### 3.2.4. DNMT Inhibition Assay Results

The inhibition of DNA methyltransferase (DNMT) activity was investigated using the colorimetric assay as described in Section 2.3. in the presence of five different compounds. Optical Density (OD) was set to 450 nm and the percentage inhibition of DNMT activity was calculated using the following formula:

$$Inhibition\%=\left[1-\frac{Inhibitor\;sample\;OD-Blank\;OD}{No\;inhibitor\;sample\;OD-Blank\;OD}\right]\times 100$$

**Figure 7:**
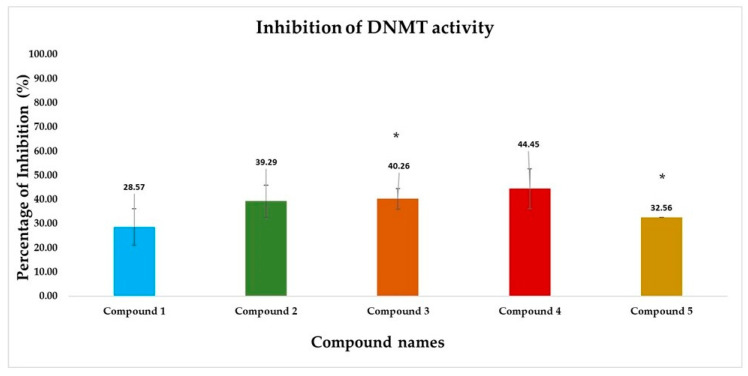
Bar chart representing the percentage inhibition of DNA methyltransferase (DNMT) activity by vs. derived hits. Each color-coded bar represents a different compound. Significant differences (*p* < 0.05) in DNMT inhibition activity between untreated control (0% inhibition) and in the presence of inhibitor denoted by asterisks (*). Compound names: **1**: 2-(3-(3,4-dimethoxyphenyl)-3-(2-((2-oxo-2H-chromen-7-yl)oxy)acetamido)propanamido)acetic acid, **2**: Phlorizin, **3**: Orientin, **4**: Bergenin, and **5**: 2-[(7,8-dihydroxy-6-undecylphenazin-2-yl)formamido]pentanedioic acid.

*t*-test analysis was performed between no inhibitor (control) and inhibitor-treated groups (compounds) ODs to assess whether the studied compounds have a significant effect on DNMT activity. The results are shown in Figure 7.

According to the results, compounds **2**, **3**, and **4** have shown better inhibition activity, while only compounds **3** and **5** have presented statistically significant differences compared with the baseline DNMT activity. It has to be noted however that *p*-values should be considered consciously since only two replicates were performed.

## 4. Discussion

According to the up-to-date literature, there are almost seventy ongoing DNMT development projects worldwide, including nucleoside and non-nucleoside DNMT inhibitors [13,45]. However, nucleoside analogs exhibit high toxicity, limited selectivity, and reduced bioavailability. Therefore, the discovery of novel non-nucleoside inhibitors has gained significant interest in the pharmaceutical landscape [13,46].

In light of the significance of epigenetic targets at the forefront of the pharmaceutical landscape, our study aimed to discover novel inhibitors sourced from natural origins that target DNA methyltransferases (DNMTs). Employing an integrated pipeline of structural and ligand-based computational approaches, a virtual screening methodology was conducted based on pharmacophore models, coupled with molecular docking and molecular dynamics simulations to establish a precise and robust selection process. Subsequently, the top-ranked compounds were procured and subjected to biological evaluation using the colorimetric DNMT activity assay (ab113467). The observed inhibition of DNA methyltransferase activity ranged from 30–45% at a concentration of 100 µM for the tested compounds.

Our in silico pipeline prioritized five natural compounds categorized into different groups, such as coumarins, flavones, chalcones, benzoic acids, and phenazine derivatives. Compound **1**, a coumarin derivative, displayed a satisfactory binding affinity due to the formation of hydrogen bonds and pi-pi interactions with Asn1578 and Phe1145 in the catalytic site of *h*DNMT1, such as Sinefungin [36]. The significance of coumarin as a scaffold for the design of DNMT1 inhibitors is proven by the fact that a series of coumarin derivatives have been proposed as DNMT1 inhibitors using a systematic computational screening protocol [47]. Interestingly, the binding free energy (ΔG_bind_) of *h*DNMT1-compound **1** complex as calculated by MM-GBSA emerged with the higher value among the derived hits in line with the displayed moderate inhibitory activity (28.57%). Compounds **2** (phlorizin), **3** (orientin), and **4** (bergenin) presented the highest, and therefore, of greater interest, inhibition potency values, approximately 40–45%. It has been hypothesized that these compounds, bearing long scaffolds, occupy the catalytic site and SAM’s cavity [45]. Also, these compounds display a common interaction pattern that can potentially explain their similar inhibitory activity (Figure 5 and Figure 6). It is critical to note that flavones, and especially genistein could inhibit the activity of DNMT1 and DNMT3 isoforms [48], reinforcing the validity of our virtual screening results. Moreover, it has to be noted that compounds **3** and **4** displayed the highest conformational stability within the active site of DNMT1 as revealed by the MD simulation. Lastly, compound **5**, a phenazine derivative, despite its fruitful interaction pattern with crucial amino acids of the *h*DNMT1 catalytic site [36,49], possessed moderate inhibitory activity potentially attributed to its higher conformational mobility within the active site as shown by MD simulations.

## 5. Conclusions

In conclusion, the current study provides valuable insights into the discovery of novel *h*DNMT inhibitors sourced from natural compounds. By employing an integrated computational and experimental approach, the study identified promising candidates for further development as potential therapeutics targeting epigenetic dysregulation. The findings contribute to advancing the field of epigenetics and drug discovery, with implications for the development of innovative treatments for diseases associated with aberrant DNA methylation patterns. Particularly, pharmacophore-based virtual screening was employed, followed by molecular docking and molecular dynamics simulations to establish a precise and reliable selection strategy. Our screening approach prioritized five naturally occurring compounds, exhibiting chemically diverse scaffolds compared to FDA-approved “Epi-drugs”. The total DNMT inhibitory activity evaluation revealed promising results for the identified hits, with inhibitory activity ranging from 30% to 45% at a concentration of 100 µM for the tested compounds. Since the examined compounds possess completely different chemical structures from known “Epi-drugs” and these structures are chemically modifiable, they could serve as starting points for further hit to lead optimization process with emphasis on the scaffold of compounds **3** and **4**.

## Figures and Tables

**Table 1:** ROC curve performance and calculated statistical significance variables values of the created pharmacophore model.

ROC Curve Performance (100% of the Screening)			
Sensitivity (Se)	0.32	Enrichment Factor (EF)	10.7
False Positive Rate (1-Sp)	0.02	Area Under the Curve (AUC)	0.65
Calculated Statistical Significance Variables Values			
^1^T	4954	^5^H_T_	148
^2^A	91	^6^H_A_	29
^3^I	14	^7^H_I_	1
^4^D	4849	^8^H_D_	118

The total number of compounds in the database ^1^(T) and the number of ^2^(A): actives, ^3^(I): inactives and ^4^(D): decoys in the database. ^5^(H_T_), ^6^(H_A_), ^7^(H_I_), and ^8^(H_D_) report the number of hits retrieved, of actives, inactives, and decoys in the hit list, respectively.

## Data Availability

Data are contained within the article and Supplementary Materials.

## Supplementary Materials

The following supporting information can be downloaded at https://www.mdpi.com/article/10.3390/cimb46040213/s1, Figure S1: RMSD calculations (50 ns) for (**A**) all Ca enzyme atoms (blue) in *h*DNMT1-Sinefungin complex and for all atoms of Sinefungin (red), (**B**) all Ca enzyme atoms (blue) in *h*DNMT1-Compound **1** complex and for all atoms of Compound **1** (red), (**C**) all Ca enzyme atoms (blue) in *h*DNMT1-Compound **2** complex and for all atoms of Compound **2** (red), (**D**) all Ca enzyme atoms (blue) in *h*DNMT1-Compound **2** complex and for all atoms of Compound **3** (red), (**E**) all Ca enzyme atoms (blue) in *h*DNMT1-Compound **4** complex and for all atoms of Compound **4** (red), (**F**) all Ca enzyme atoms (blue) in *h*DNMT1-Compound **5** complex and for all atoms of Compound **5** (red).; Figure S2: RMSF calculations of ligand atomic positions throughout molecular dynamics simulations (50 ns) of (A) Sinefungin, (B) Compound **1**, (C) Compound **2**, (D) Compound **3**, (E) Compound **4** and (F) Compound **5** in complex with *h*DNMT1; Table S1: Chemical structures and class of the training set compounds; Table S2: Chemical structures and class of the test set compounds; Table S3: Predicted physicochemical parameters of epigenetic drugs; Table S4: Docking scores and interaction pattern of the most promising compounds; Table S5: Predicted ADMET properties of the most promising compounds, using ADMETlab 2.0 open source software; Table S6: Binding energy (kcal · mol-1) and individual energy terms of *h*DNMT1-selected compounds complexes calculated, using Prime MM-GBSA.
