# Supplementary material for: A Computational Approach for the Discovery of Novel DNA Methyltransferase Inhibitors
Source: Curr Issues Mol Biol. 2024 Apr 16;46(4):3394–407. doi: 10.3390/cimb46040213 (PMC11049320; doi:10.3390/cimb46040213)
Supplement: Supplementary file 1 [file cimb-46-00213-s001.zip › cimb-2902138-supplementary.pdf]

A Computational Approach for the Discovery of Novel DNA Methyltransferase Inhibitors

Eftichia Kritsi *, Paris Christodoulou, Thalia Tsiaka, Panagiotis Georgiadis and Maria Zervou *

Institute of Chemical Biology, National Hellenic Research Foundation, 48 Vassileos Constantinou Avenue, 11635 Athens, Greece; pchristodoulou@eie.gr (P.C.); thtsiaka@eie.gr (T.T.); panosg@eie.gr (P.G.)

* Correspondence: ekritsi@eie.gr (E.K.); mzervou@eie.gr (M.Z.)

Table S1: Chemical structures and class of the training set compounds

Structure	Name	Class
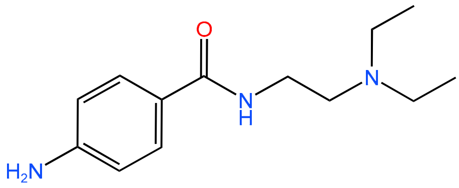	Procainamide	DNMT1 (IC_{50} = 300 uM) inhibitor
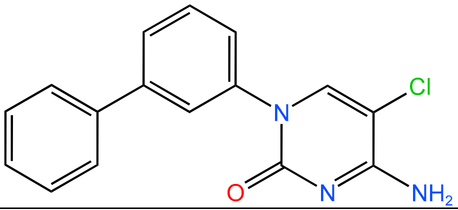	Bobcat339	TET1 (IC_{50} = 33 uM) & TET2 (IC_{50} = 73 uM) Inhibitor
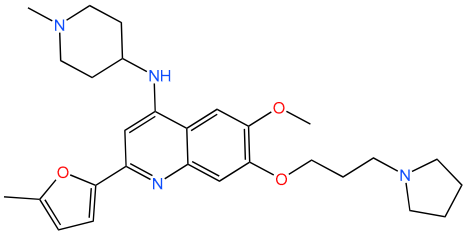	CM272	DNMT1 inhibitor (IC_{50} = 382 nM)
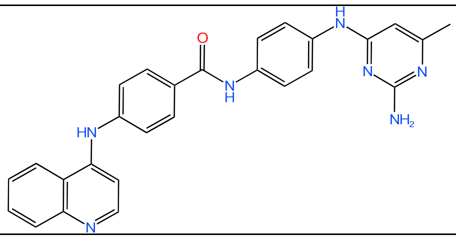	SGI-1027	DNMT1 (IC_{50} = 12.5 uM) & DNMT3A (IC_{50} = 8 uM) & DNMT3B (IC_{50} = 7.5 uM) inhibitor
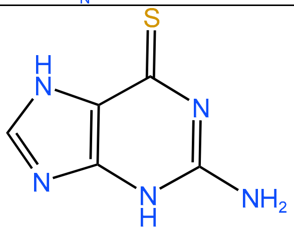	Thioguanine	DNMT1 inhibitor (inhibits DNMT1 activity through ubiquitin-targeted degradation, used in the treatment of acute lymphoblastic leukemia, autoimmune disorders (e.g., Crohn's disease,

		rheumatoid arthritis) and organ transplant recipients
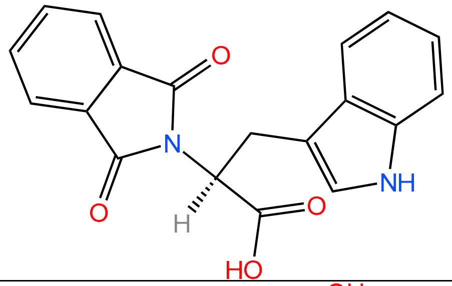	RG108	DNMT (IC_{50} = 115 nM) inhibitor
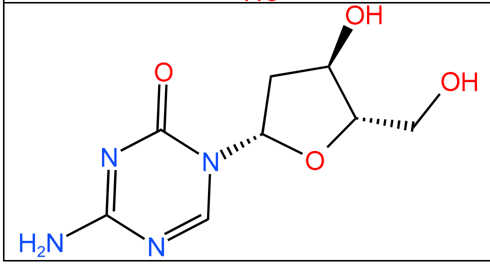	Decitabine	DNMT (IC_{50} = 30 nM) inhibitor

[1] All activity values are retrieved from ChEMBL database (https://www.ebi.ac.uk/chembl/) and Selleck Chem (https://www.selleckchem.com)

Table S2: Chemical structures and class of the test set compounds

Structure	Name	Class
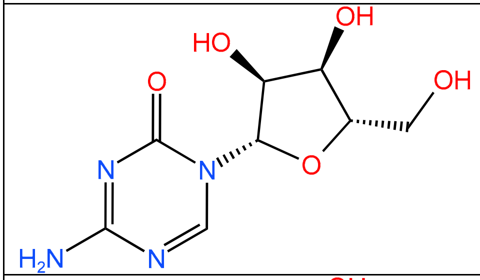	Azacitidine	DNMT1 inhibitor
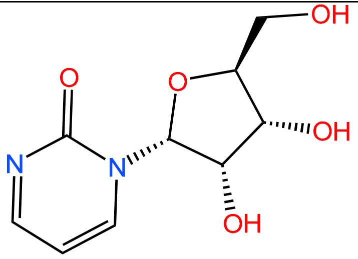	Zebularine	DNMT inhibitor
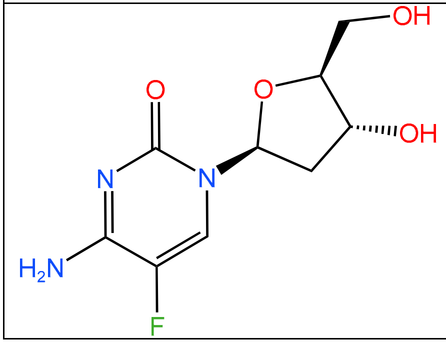	2'-Deoxy-5-Fluorocytidine	DNMT inhibitor

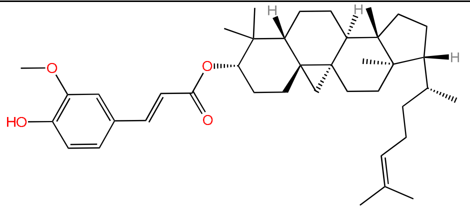	Gamma-Oryzano	DNMT1 & DNMT3A inhibitor
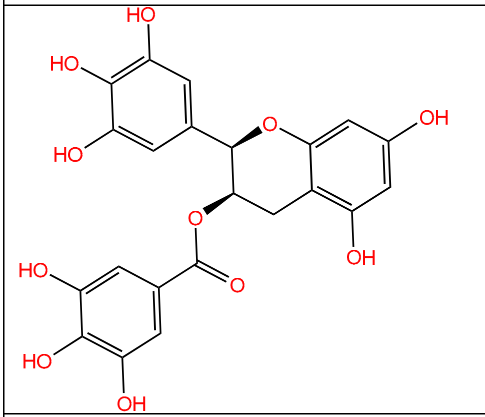	(-)-Epigallocatechin gallate	DNMT1 & HDAC1 inhibitor
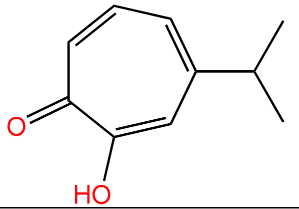	β-thujaplicin	DNMT1 inhibitor

Table S3: Predicted physicochemical parameters of epigenetic drugs.

Compounds	AlogP[1]	MW[2]	HBA[3]	HBD[4]	RB[5]	PSA[6]
Decitabine	-0.5171	228.2053	6	3	2	123.5
Azacitidine	-1.2834	244.2047	7	4	2	143.7
Procainamide	1.1278	235.3253	1	2	6	58.36
Hydralazine	0.6187	160.1759	2	2	1	63.83
Romidepsin	1.6898	540.6958	6	4	2	193.3
Panobinostat	2.6833	349.4262	2	3	7	77.15
Belinostat	1.6683	318.3477	4	3	5	103.9
Vorinostat	1.9802	264.3202	3	3	8	78.43

[1] AlogP: lipophilicity, [2] MW: Molecular Weight, [3] HBA: Number of Hydrogen bond-Acceptors, [4] HBD: Number of Hydrogen bond-Donors, [5] RB: Rotatable Bonds and [6] PSA: Polar Surface Area

Table S4: Docking scores and interaction pattern of the most promising compounds.

**Compounds**	**Docking score (kcal·mol^{-1})**			**Interaction pattern**		
PDB: 3SWR	Glide-SP	Glide-XP	IFD	Glide-SP	Glide-XP	IFD
Sinefungin (crystal structure)	-10.60	-10.20	-11.93	HB Phe1145, HB, Leu1151, HB Ile1167, HB Glu1168, HB Met1169, HB Asp1190, HB Cys1191, HB Asn1578, HB Val1580		
1	-8.99	-9.68	-11.63	WB Asp1143	pi-pi/HB **Phe1145**	HB/WB **Asp1143**

				HB/WB<br>**Phe1145**<br>HB **Met1169**<br>WB **Asn1578**<br>HB **Val1580**	WB Cys1148<br>HB Gly1149<br>HB Gly1150<br>Leu**1151**<br>HB **Met1169**<br>WB **Asn1578**	HB/WB<br>**Phe1145**<br>HB Gly1150<br>HB **Cys1191**<br>WB **Asn1578**<br>HB **Val1580**
2	-7.69	-10.44	-9.41	HB Glu698<br>HB **Glu1168**<br>HB **Asp1190**<br>WB **Asn1578**	HB/pi-pi<br>**Phe1145**<br>HB **Glu1168**<br>HB **Asp1190**<br>HB Gly1223	HB Glu698<br>HB Ala699<br>pi-pi **Phe1145**<br>WB Trp1170<br>HB **Cys1191**<br>HB Gly1223<br>HB Gln1227<br>WB **Asn1578**
3	-7.80	-11.32	-9.82	HB Glu698<br>HB **Glu1168**<br>HB **Asp1190**<br>HB Gln1227<br>WB **Asn1578**	HB Glu698<br>HB **Glu1168**<br>HB **Asp1190**<br>WB **Asn1578**	HB **Ile1167**<br>HB **Glu1168**<br>HB **Asp1190**<br>HB Gln1227<br>HB Arg1574<br>WB **Asn1578**
4	-7.96	-9.93	-8.15	HB **Glu1168**<br>HB **Met1169**<br>HB **Asp1190**<br>WB **Asn1578**	HB **Glu1168**<br>HB **Met1169**<br>HB **Asp1190**<br>WB **Asn1578**	HB Ala699<br>HB Asn700<br>HB **Phe1145**<br>HB Glu1266<br>HB Arg1312<br>HB **Asn1578**
5	-8.45	-9.78	-10.73	HB Ser1146<br>HB Gly1150<br>HB **Leu1151**<br>pi-pi Trp1170<br>HB Glu1266<br>HB **Val1580**	HB/pi-pi<br>**Phe1145**<br>HB Ser1146<br>WB Cys1148<br>HB Gly1150<br>HB **Asp1190**<br>HB Glu1266<br>HB **Val1580**	HB Asn700<br>HB/WB<br>**Phe1145**<br>HB **Cys1190**<br>HB Glu1266<br>HB Arg1312<br>HB **Asn1578**
1 (COO^-)	-7.92	-4.02	-9.33	pi-pi **Phe1145**<br>WB Cys1148<br>HB Gly1149<br>HB Gly1150<br>HB **Met1169**<br>WB **Asn1578**<br>HB **Val1580**	WB Cys1148<br>HB Gly1149<br>HB Gly1150<br>HB **Leu1151**<br>HB **Met1169**<br>WB **Asn1578**	HB Asn700,<br>HB/WB<br>**Phe1145**<br>WB Cys1148<br>HB Gly1149<br>HB Gly1150<br>HB **Leu1151**<br>WB **1578**
5 (COO^-)	-4.05	-2.24	-7.50	WB Cys1148<br>pi-pi Trp1170<br>HB **Val1580**	pi-pi **Phe1145**<br>WB Cys1148<br>HB **Asp1190**<br>SB Arg1310<br>WB **Asn1578**	SB Lys629<br>pi-pi **Phe1145**<br>HB **Glu1168**<br>HB Gln1227

WB: water bridge, HB: hydrogen bond, pi-pi: pi-pi interactions.

Table S5: Predicted ADMET properties of the most promising compounds, using ADMETlab 2.0 open source software.

	Physicochemical							Medicinal Chemistry			Absorption			Distribution	
**Compound**	logP[1]	MW[2]	HBA[3]	HBD[4]	RB[5]	TPSA[6]	logS[7]	Lipinski rule[8]	Pfizer rule[9]	GSK rule[10]	Caco-2 permeability[11]	MDCK permeability[12]	HIA[13]	PPB[14]	BBB[15]
**1**	0.623	484.15	11	3	13	153.4	-3.107	🟢	🟢	🔴	🔴	🟢	🟢	🟢	🟢
**2**	0.509	436.14	10	7	7	177.14	-2.21	🟢	🟢	🔴	🔴	🟢	🔴	🟢	🟢
**3**	0.757	448.10	11	8	3	201.28	-3.697	🔴	🟢	🔴	🔴	🟢	🔴	🟢	🟢
**4**	-0.285	328.08	9	5	2	145.91	-1.508	🟢	🟢	🟢	🔴	🟢	🔴	🟢	🟠
**5**	5.352	539.26	10	5	17	169.94	-3.526	🔴	🟢	🔴	🔴	🟢	🟢	🔴	🟢

[1] logP: The logarithm of the n-octanol/water distribution coefficient. The predicted logP of a compound is given as the logarithm of the molar concentration (log mol/L). Compounds in the range from 0 to 3 log mol/L will be considered proper, [2] MW: Molecular Weight (Optimal:100~600, based on Drug-Like Soft rule), [3] HBA: Number of Hydrogen bond-Acceptors (Optimal: 0~12, based on Drug-Like Soft rule), [4] HBD: Number of Hydrogen bond-Donors (Optimal: 0~7, based on Drug-Like Soft rule), [5] RB: Number of Rotatable Bonds (Optimal: 0~11, based on Drug-Like Soft rule), [6] TPSA: Topological Polar Surface Area (Optimal:0~140, based on Veber rule), [7] logS: The logarithm of aqueous solubility value. The predicted solubility of a compound is given as the logarithm of the molar concentration (log mol/L). Compounds in the range from -4 to 0.5 log mol/L will be considered proper, [8] Lipinski rule: Empirical decision: <2 violations ： excellent (green) ; ≥2 violations: poor (red) absorption or permeability is possible, [9] Pfizer rule: Compounds with a high log P (>3) and low TPSA (<75) are likely to be toxic. Empirical decision: two conditions satisfied: poor (red); otherwise: excellent (green), [10] GSK rule: Compounds satisfying the GSK rule may have a more favorable ADMET profile. Empirical decision: 0 violations: excellent (green); otherwise: poor (red), [11] Caco-2 permeability: The predicted Caco-2 permeability of a given compound is given as the log cm/s. A compound is considered to have a proper Caco-2 permeability if it has predicted value >-5.15log cm/s. Empirical decision: > -5.15: excellent (green); otherwise: poor (red), [12] MDCK permeability: Empirical decision: >2 x 10^{-6} cm/s: excellent (green), otherwise: poor (red), [13] HIA: A molecule with an absorbance of less than 30% is considered to be poorly absorbed. Accordingly, molecules with a HIA >30% were classified as HIA- (Category 0), while molecules with a HIA < 30% were classified as HIA+(Category 1). The output value is the probability of being HIA+, within the range of 0 to 1. Empirical decision: 0-0.3: excellent (green); 0.3-0.7: medium (yellow); 0.7-1.0(++): poor (red), [14] PPB: A compound is considered to have a proper Plasma Protein Binding if it has predicted value < 90%, and drugs with high protein-bound may have a low therapeutic index. Empirical decision: ≤ 90%: excellent (green); otherwise: poor (red), [15] BBB: The unit of BBB penetration is cm/s. Molecules with logBB > -1 were classified as BBB+ (Category 1), while molecules with logBB ≤ -1 were classified as BBB- (Category 0). The output value is the probability of being BBB+, within the range of 0 to 1. Empirical decision: 0-0.3: excellent (green); 0.3-0.7: medium (yellow); 0.7-1.0(++): poor (red).

Compound	Metabolism					Excretion
**Compound**	CYP1A2 inhibitor[16]	CYP2C19 inhibitor[17]	CYP2C9 inhibitor[18]	CYP2D6 inhibitor[19]	CYP3A4 inhibitor[20]	CL[21]
**1**	0.022	0.087	0.116	0.012	0.041	🟢
**2**	0.392	0.063	0.055	0.608	0.192	🟢
**3**	0.199	0.020	0.071	0.028	0.058	🟢
**4**	0.057	0.015	0.004	0.008	0.016	🟢
**5**	0.157	0.061	0.444	0.076	0.029	🔴

[16-20] Based on the chemical nature of biotransformation, the process of drug metabolism reactions can be divided into two broad categories: phase I (oxidative reactions) and phase II (conjugative reactions). The human cytochrome P450 family (phase I enzymes) contains 57 isozymes and these isozymes metabolize approximately two-thirds of known drugs in human with 80% of this attribute to five isozymes—1A2, 3A4, 2C9, 2C19 and 2D6. Most of these CYPs responsible for phase I reactions are concentrated in the liver. Category 0: Non-inhibitor; Category 1: inhibitor. The output value is the probability of being inhibitor, within the range of 0 to 1, [21] CL: Clearance is an important pharmacokinetic parameter that defines, together with the volume of distribution, the half-life, and thus the frequency of dosing of a drug. The unit of predicted CL penetration is ml/min/kg. >15 ml/min/kg: high clearance; 5-15 ml/min/kg: moderate clearance; <5 ml/min/kg: low clearance. Empirical decision: ≥ 5: excellent (green);< 5: poor (red).

Toxicity									
**Compound**	hERG blockers[22]	Skin sensitization[23]	Carcinogenicity[24]	Eye irritation[25]	Respiratory toxicity[26]	NR-AR LBD[27]	NR-ER LBD[28]	NR-AhR[29]	NR-PPARγ[30]
**1**	🟢	🟢	🟢	🟢	🟢	🟢	🟢	🟢	🟢
**2**	🟢	🔴	🟢	🟢	🟢	🟠	🟢	🔴	🟢
**3**	🟢	🔴	🟢	🟠	🟢	🟢	🔴	🔴	🟠
**4**	🟢	🟠	🟢	🟢	🟢	🟢	🟢	🟠	🟢
**5**	🟢	🟠	🟢	🟢	🔴	🟠	🟢	🔴	🔴

[22] hERG blockers: Molecules with IC50 more than 10 μM or less than 50% inhibition at 10 μM were classified as hERG - (Category 0), while molecules with IC50 less than 10 μM or more than 50% inhibition at 10 μM were classified as hERG+ (Category 1). The output value is the probability of being hERG+, within the range of 0 to 1. Empirical decision: 0-0.3: excellent (green); 0.3-0.7: medium (yellow); 0.7-1.0(++): poor (red), [23] Skin sensitizations: Category 1: Sensitizer; Category 0: Non-sensitizer. The output value is the probability of being toxic, within the range of 0 to 1. Empirical decision: 0-0.3: excellent (green); 0.3-0.7: medium (yellow); 0.7-1.0(++): poor (red), [24] Carcinogenicity: Category 1: carcinogens; Category 0: non-carcinogens. Chemicals are labelled as active (carcinogens) or inactive (non-carcinogens) according to their TD50 values. The output value is the

probability of being toxic, within the range of 0 to 1. Empirical decision: 0-0.3: excellent (green); 0.3-0.7: medium (yellow); 0.7-1.0(++): poor (red), [25] Eye irritation: Category 1: irritants chemicals; Category 0: non-irritants chemicals. The output value is the probability of being toxic, within the range of 0 to 1. Empirical decision: 0-0.3: excellent (green); 0.3-0.7: medium (yellow); 0.7-1.0(++): poor (red), [26] Respiratory toxicity: Category 1: respiratory toxicants; Category 0: non-respiratory toxicants. The output value is the probability of being toxic, within the range of 0 to 1. Empirical decision: 0-0.3: excellent (green); 0.3-0.7: medium (yellow); 0.7-1.0(++): poor (red), [27] NR-AR LBD: Androgen receptor (AR), a nuclear hormone receptor, plays a critical role in AR-dependent prostate cancer and other androgen related diseases. Endocrine disrupting chemicals (EDCs) and their interactions with steroid hormone receptors like AR may cause disruption of normal endocrine function as well as interfere with metabolic homeostasis, reproduction, developmental and behavioral functions. Category 1: actives; Category 0: inactives. Molecules that labeled 1 in this bioassay may bind to the LBD of androgen receptor. The output value is the probability of being actives, within the range of 0 to 1. Empirical decision: 0-0.3: excellent (green); 0.3-0.7: medium (yellow); 0.7-1.0(++): poor (red), [29] NR-AhR: The Aryl hydrocarbon Receptor (AhR), a member of the family of basic helix-loop-helix transcription factors, is crucial to adaptive responses to environmental changes. AhR mediates cellular responses to environmental pollutants such as aromatic hydrocarbons through induction of phase I and II enzymes but also interacts with other nuclear receptor signaling pathways. Category 1: actives; Category 0: inactives. Molecules that labeled 1 may activate the aryl hydrocarbon receptor signaling pathway. The output value is the probability of being actives, within the range of 0 to 1. Empirical decision: 0-0.3: excellent (green); 0.3-0.7: medium (yellow); 0.7-1.0(++): poor (red), [30] NR-PPARγ: The peroxisome proliferator-activated receptors (PPARs) are lipid-activated transcription factors of the nuclear receptor superfamily with three distinct subtypes namely PPAR alpha, PPAR delta (also called PPAR beta) and PPAR gamma (PPARg). All these subtypes heterodimerize with Retinoid X receptor (RXR) and these heterodimers regulate transcription of various genes. PPAR-gamma receptor (glitazone receptor) is involved in the regulation of glucose and lipid metabolism. Category 1: actives; Category 0: inactives. The output value is the probability of being actives within the range of 0 to 1. Empirical decision: 0-0.3: excellent (green); 0.3-0.7: medium (yellow); 0.7-1.0(++): poor (red).

Table S6. Binding energy (kcal · mol^{-1}) and individual energy terms of *h*DNMT1-selected compounds complexes calculated, using Prime MM-GBSA.

**Compound**	**ΔG_{Bind}[1] (kcal·mol-1)**	**$\Delta G_{Coulomb}$[2] (kcal·mol^{-1})**	**ΔG_{Hbond}[3] (kcal·mol^{-1})**	**ΔG_{Lipo}[4] (kcal·mol^{-1})**	**ΔG_{Solv}[5] (kcal·mol^{-1})**	**ΔG_{vdW}[6] (kcal·mol^{-1})**
Sinefungin	-95.03	-48.58	-6.84	-23.47	40.65	-62.04
1	-42.87	13.86	-7.69	-35.67	-16.74	-12.42
2	-69.84	-31.35	-3.01	-35.86	38.43	-43.57
3	-68.19	-27.10	-3.78	-34.58	38.75	-44.56
4	-65.12	-22.50	-4.03	-36.47	37.95	-44.56
5	-95.97	-48.93	-2.68	-51.80	50.16	-59.11

* [1]ΔG_{Bind}: total enegy, [2]$\Delta G_{Coulomb}$: Coulomb energy, [3]ΔG_{Hbond}: Hydrogen-bonding correction energy, [4]ΔG_{Lipo}: Lipophilic energy, [5]ΔG_{Solv}: Generalized Born electrostatic solvation energy, [7]ΔG_{vdW}: Van der Waals energy

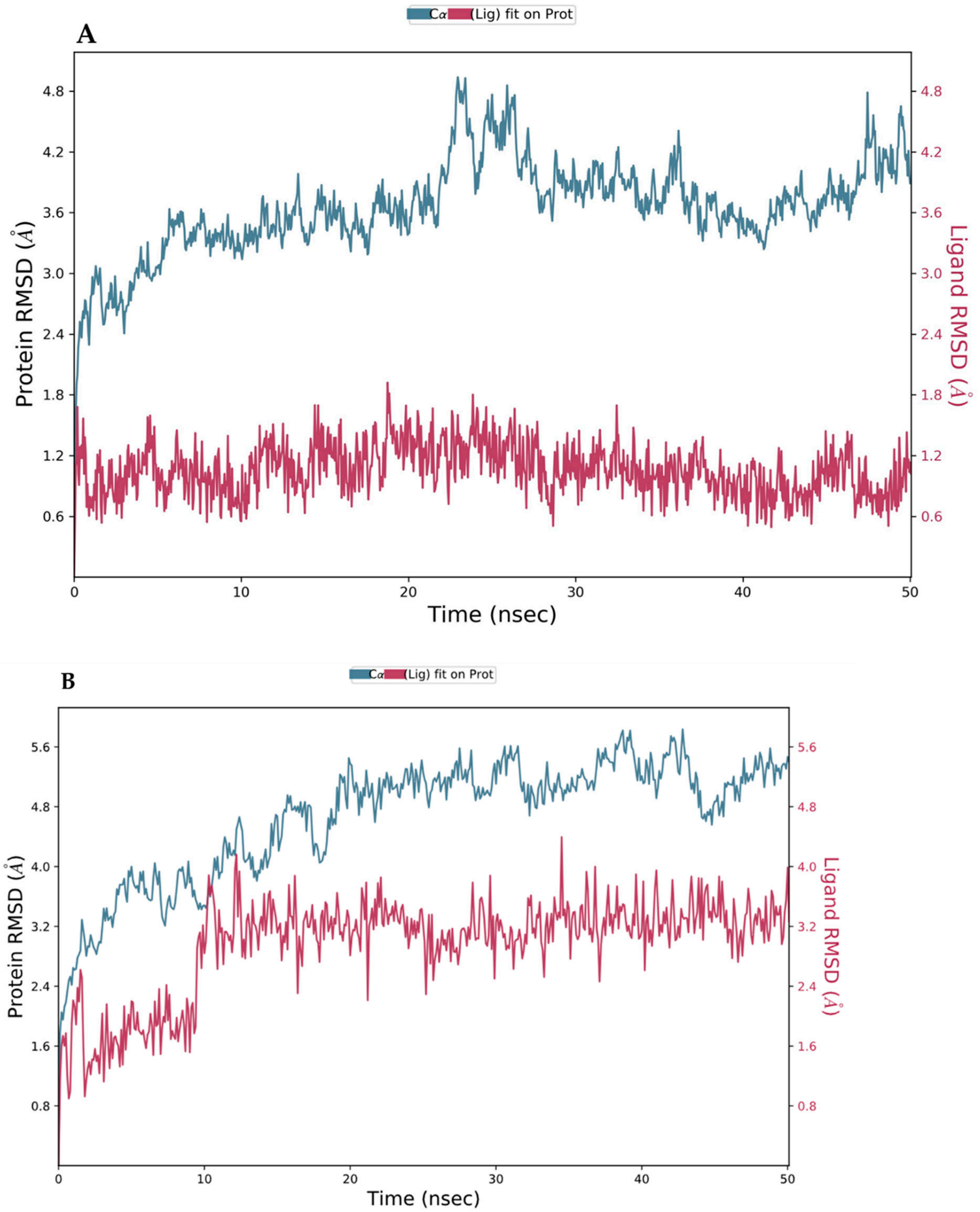

A
Cα (Lig) fit on Prot
Protein RMSD (Å)
Ligand RMSD (Å)
Time (nsec)
4.8
4.2
3.6
3.0
2.4
1.8
1.2
0.6
0
10
20
30
40
50
B
Cα (Lig) fit on Prot
Protein RMSD (Å)
Ligand RMSD (Å)
Time (nsec)
5.6
4.8
4.0
3.2
2.4
1.6
0.8
0
10
20
30
40
50

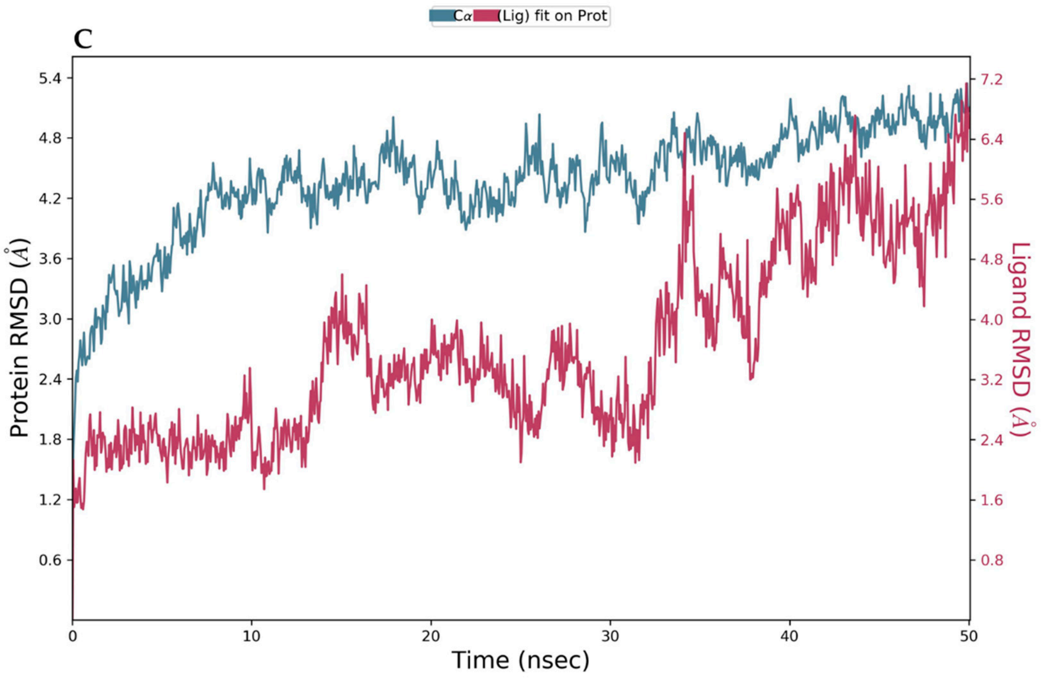
Cα (Lig) fit on Prot
C
Protein RMSD (Å)
Ligand RMSD (Å)
Time (nsec)
5.4
4.8
4.2
3.6
3.0
2.4
1.8
1.2
0.6
7.2
6.4
5.6
4.8
4.0
3.2
2.4
1.6
0.8
0
10
20
30
40
50

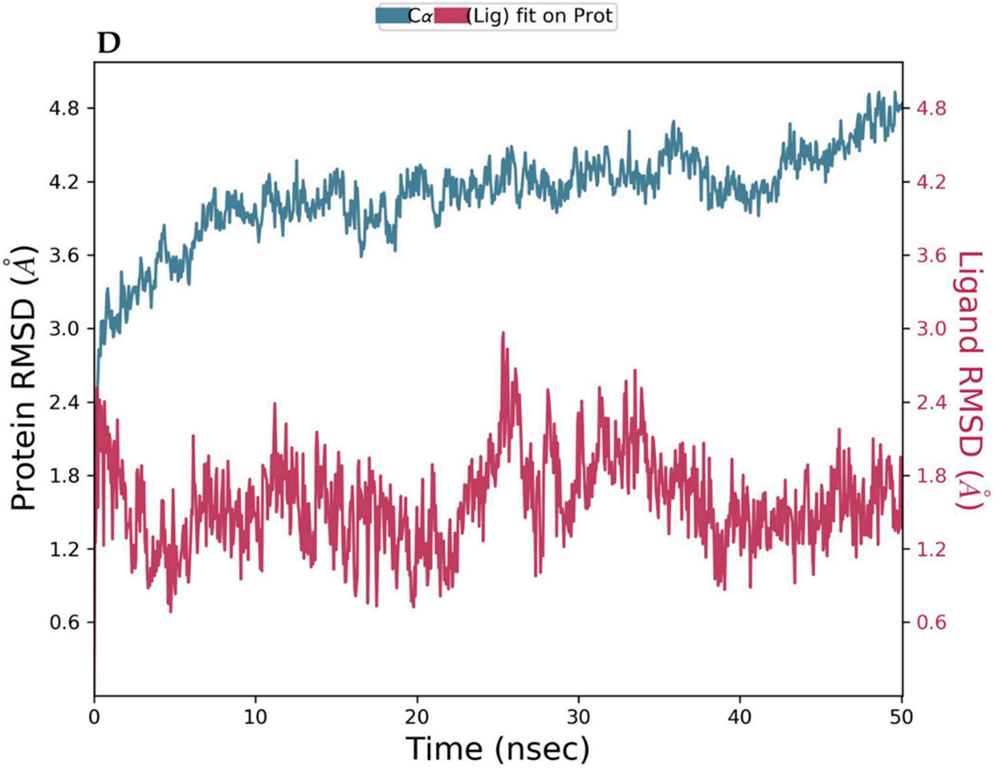
Cα (Lig) fit on Prot
D
Protein RMSD (Å)
Ligand RMSD (Å)
Time (nsec)
4.8
4.2
3.6
3.0
2.4
1.8
1.2
0.6
0
10
20
30
40
50

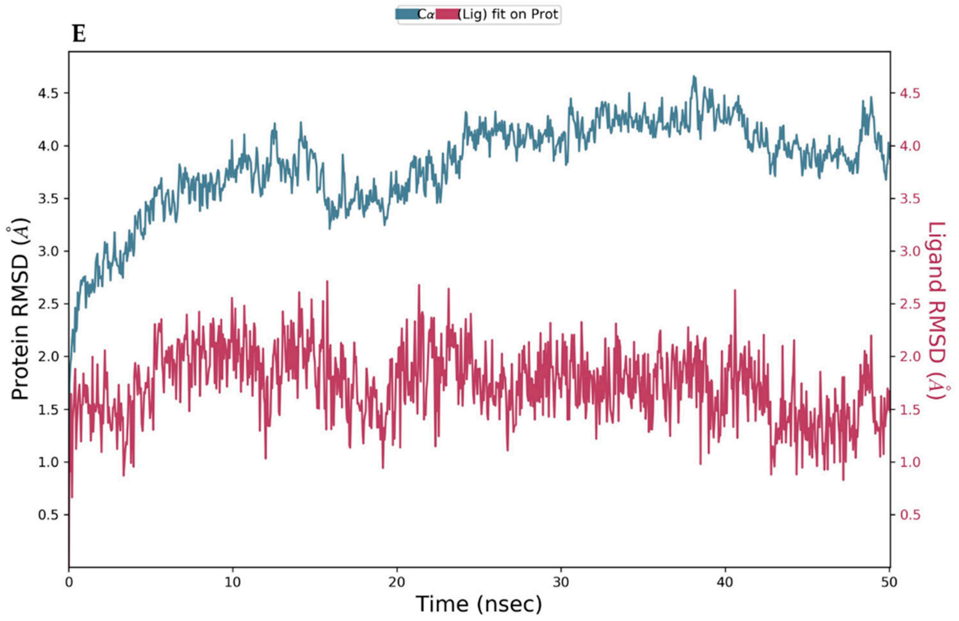

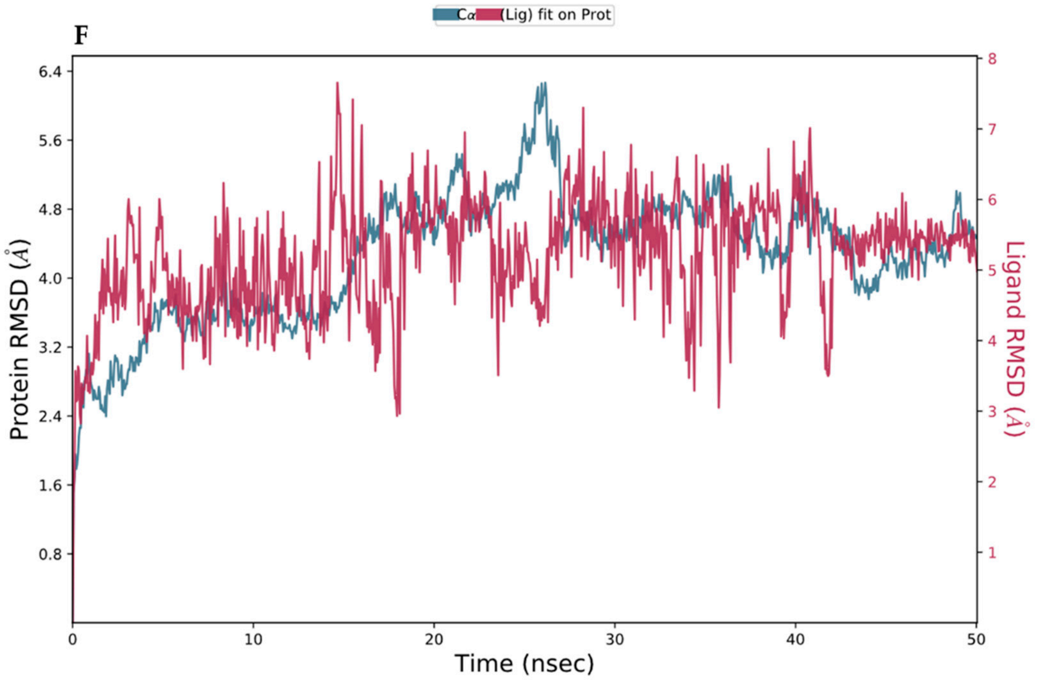

Figure S1: RMSD calculations (50 ns) for (**A**) all Ca enzyme atoms (blue) in *h*DNMT1-Sinefungin complex and for all atoms of Sinefungin (red), (**B**) all Ca enzyme atoms (blue) in *h*DNMT1-Compound 1 complex and for all atoms of Compound 1 (red), (**C**) all Ca enzyme atoms (blue) in *h*DNMT1-Compound 2 complex and for all atoms of Compound 2 (red), (**D**) all Ca enzyme atoms (blue) in *h*DNMT1-Compound 2 complex and for all atoms of Compound 3 (red), (**E**) all Ca enzyme atoms (blue) in *h*DNMT1-Compound 4 complex and for all atoms of Compound 4 (red), (**F**) all Ca enzyme atoms (blue) in *h*DNMT1-Compound 5 complex and for all atoms of Compound 5 (red).

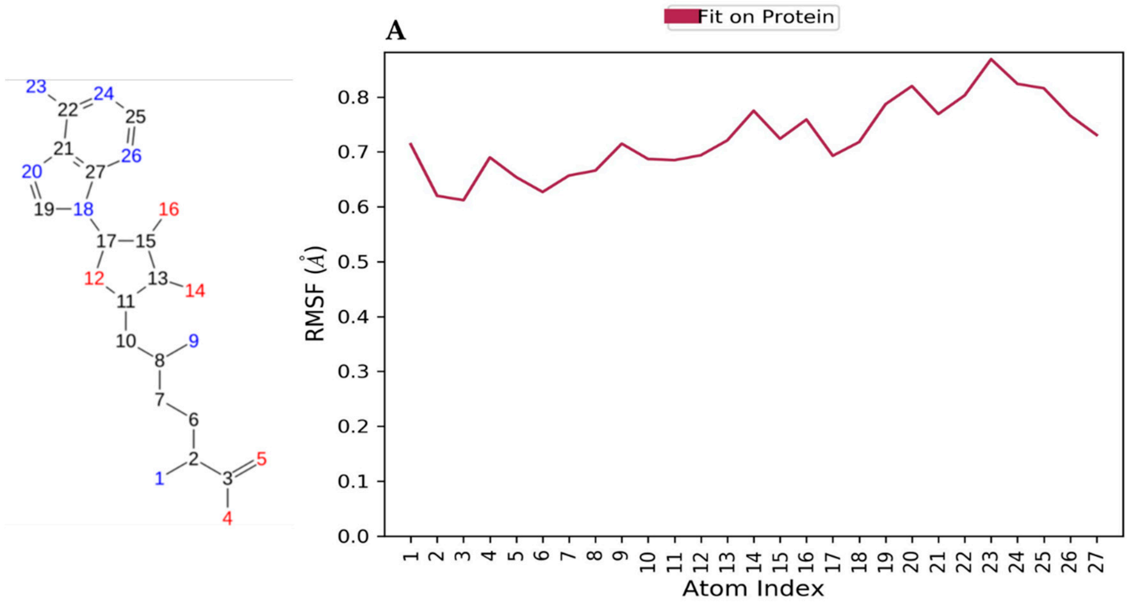
A
Fit on Protein
RMSF (Å)
Atom Index

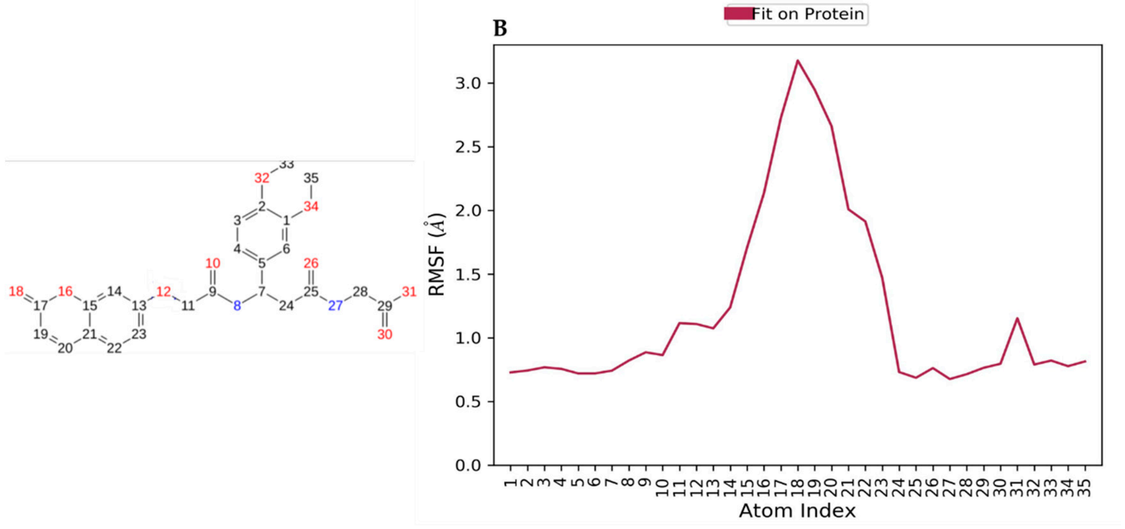
B
Fit on Protein
RMSF (Å)
Atom Index

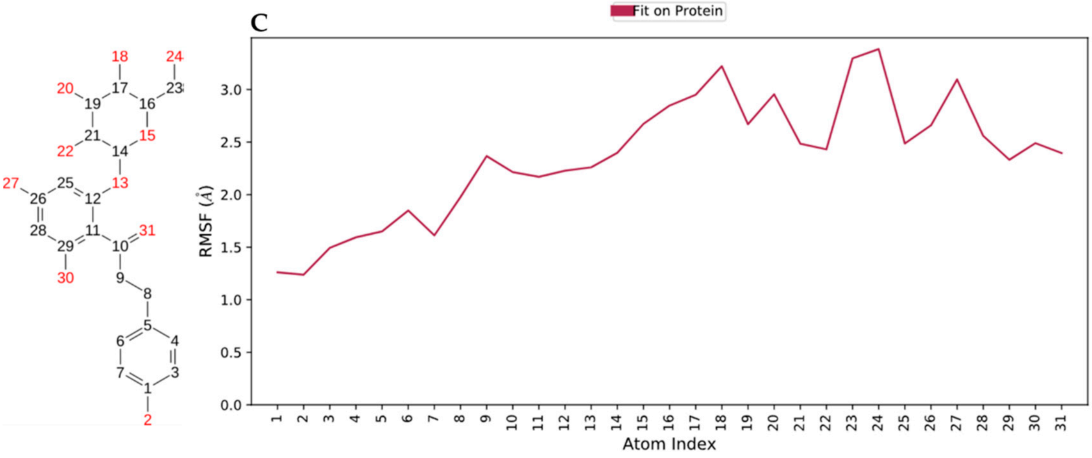
C
Fit on Protein
RMSF (Å)
Atom Index

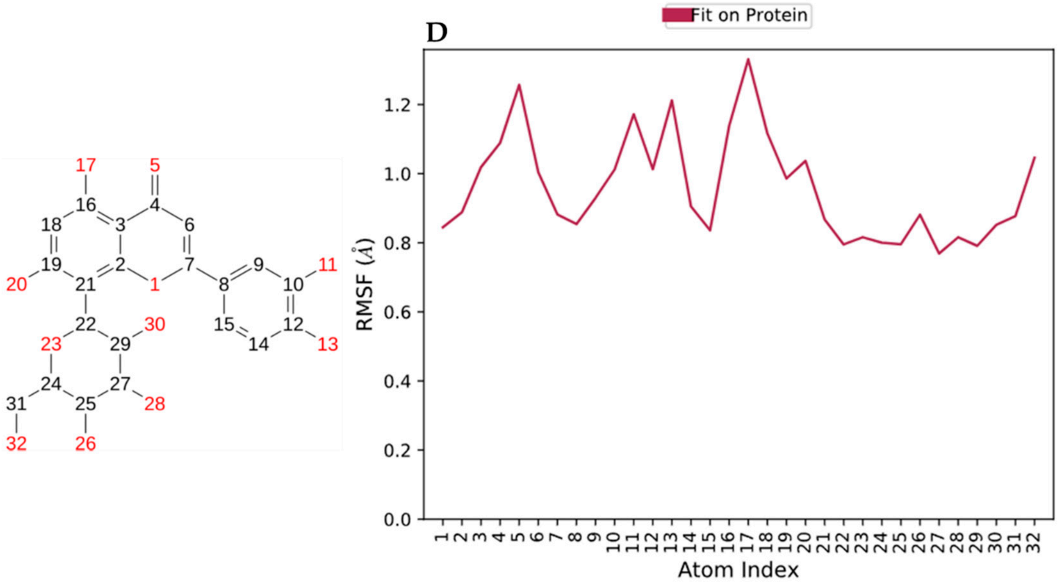
D
Fit on Protein
RMSF (Å)
Atom Index

E

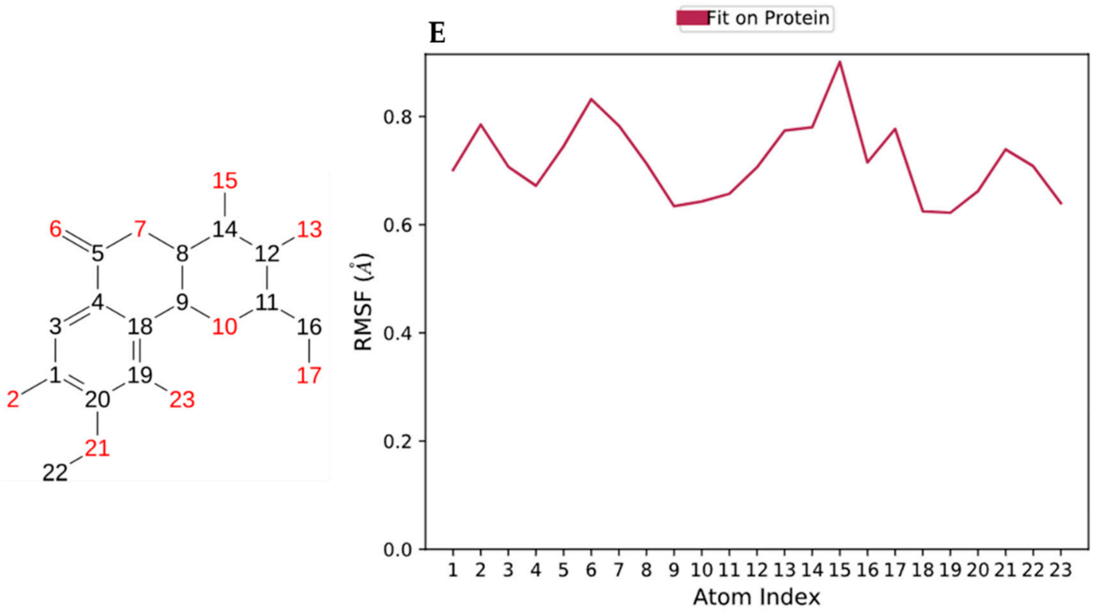

F

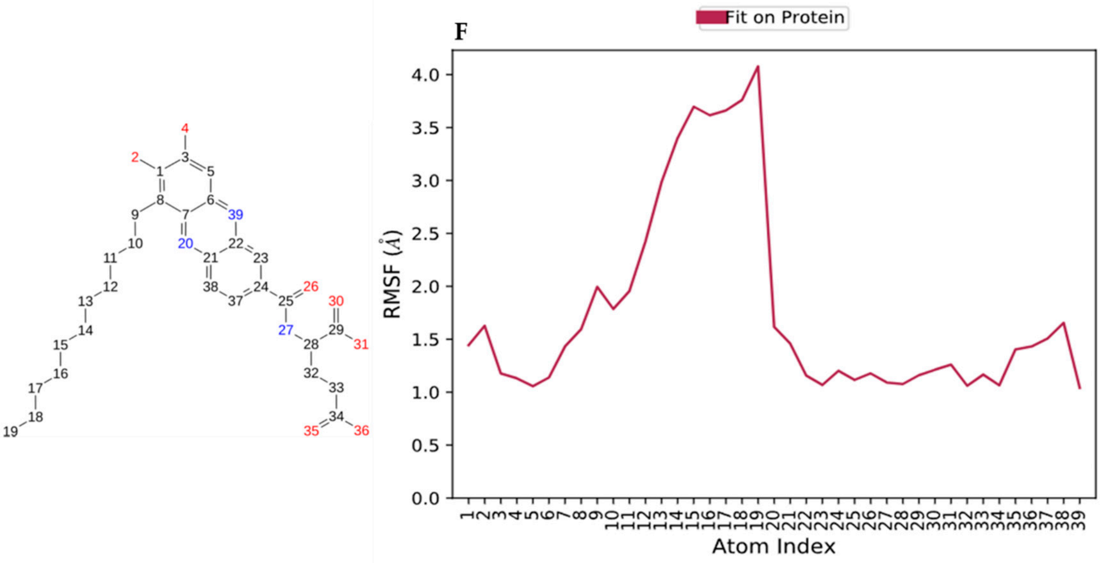

Figure S2: RMSF calculations of ligand atomic positions throughout molecular dynamics simulations (50ns) of (**A**) Sinefungin, (**B**) Compound 1, (**C**) Compound 2, (**D**) Compound 3, (**E**) Compound 4 and (**F**) Compound 5 in complex with *h*DNMT1.
